# Monomeric C-Reactive Protein Aggravates Secondary Degeneration after Intracerebral Haemorrhagic Stroke and May Function as a Sensor for Systemic Inflammation

**DOI:** 10.3390/jcm9093053

**Published:** 2020-09-22

**Authors:** Mark Slevin, Elisa García-Lara, Bogdan Capitanescu, Coral Sanfeliu, Yasmin Zeinolabediny, Raid AlBaradie, Peter Olah, Baoqiang Guo, Daniel Pirici, Mario Di Napoli, Aurel Popa-Wagner

**Affiliations:** 1 Department of Life Sciences, Faculty of Science and Engineering, Manchester Metropolitan University, Chester Street, Manchester M15 6BH, UK; m.a.slevin@mmu.ac.uk (M.S.); Yaasi_zei@icloud.com (Y.Z.); 2 The University of Medicine, Pharmacy, Science and Technology at Targu Mures, 540142 Targu Mures, Romania; olah_peter@yahoo.com; 3 Institut d’Investigacions Biomèdiques de Barcelona (IIBB), CSIC, IDIBAPS and CIBERESP, 08036 Barcelona, Spain; elisaglara21@gmail.com (E.G.-L.); coral.sanfeliu@iibb.csic.es (C.S.); 4 Department of Anatomy, University of Medicine and Pharmacy Craiova, 200349 Craiova, Romania; bogdanc26@yahoo.com; 5 Applied Medical Sciences College, Majmaah University, Al Majma’ah 15361, Saudi Arabia; r.albaradie@mu.edu.sa; 6 Regenesol LTD, Number 30 the Green Building, 19 New Wakefield Street, Manchester M1 5NP, UK; b.guo@mmu.ac.uk; 7 Department of Research Methodology, University of Medicine and Pharmacy Craiova, 200349 Craiova, Romania; danielpirici@yahoo.com; 8 Department of Neurology and Stroke Unit, San Camillo de’ Lellis General Hospital, 02100 Rieti, Italy; 9 Neurological Section, Neuro-epidemiology Unit, SMDN, Centre for Cardiovascular Medicine and Cerebrovascular Disease Prevention, Sulmona, 67039 L’Aquila, Italy; 10 Center of Eexperimental and Clinical Medicine, University of Medicine and Pharmacy, 200349 Craiova, Romania; 11 Griffith University Menzies Health Institute of Queensland, Gold Coast Campus, Gold Coast Campus, QLD 4222, Australia

**Keywords:** mCRP, haemorrhagic stroke, neuroinflammation, hypothalamus, micro-circulatory system

## Abstract

Background: We previously identified increased tissue localization of monomeric C-reactive protein (mCRP) in the infarcted cortical brain tissue of patients following ischaemic stroke. Here, we investigated the relationship of mCRP expression in haemorrhagic stroke, and additionally examined the capacity of mCRP to travel to or appear at other locations within the brain that might account for later chronic neuroinflammatory or neurodegenerative effects. Methods: Immunohistochemistry was performed on Formalin-fixed, paraffin-embedded archived brain tissue blocks obtained at autopsy from stroke patients and age-matched controls. We modelled mCRP migration into the brain after haemorrhagic stroke by infusing mCRP (3.5 µg) into the hippocampus of mice and localized mCRP with histological and immunohistochemistry methods. Results: On human tissue in the early stages of haemorrhage, there was no staining of mCRP. However, with increasing post-stroke survival time, mCRP immunostaining was associated with some parenchymal brain cells, some stroke-affected neurons in the surrounding areas and the lumen of large blood vessels as well as brain capillaries. Further from the peri-haematoma region, however, mCRP was detected in the lumen of micro-vessels expressing aquaporin 4 (AQP4). In the hypothalamus, we detected clusters of neurons loaded with mCRP along with scattered lipofuscin-like deposits. In the peri-haematoma region of patients, mCRP was abundantly seen adjacent to AQP4 immunoreactivity. When we stereotactically injected mCRP into the hippocampus of mice, we also observed strong expression in distant neurones of the hypothalamus as well as cortical capillaries. Conclusions: mCRP is abundantly expressed in the brain after haemorrhagic stroke, directly impacting the pathophysiological development of the haematoma. In addition, it may have indirect effects, where the microcirculatory system appears to be able to carry it throughout the cortex as far as the hypothalamus, allowing for long-distance effects and damage through its capacity to induce inflammation and degenerate neuronal perivascular compartments.

## 1. Introduction

Intracerebral haemorrhage (ICH) represents a major cause of morbidity and mortality worldwide [1]. General critical management of the acute event represents the only established therapy.

The frequencies of dementia and cognitive impairment after ICH have not been systematically studied [2]. Risk factors and possible etiologic determinants remain poorly understood. Larger size of the haematoma and location in the brain were associated with risk for early post-ICH dementia within six months. Educational level, mood symptoms and the severity of white matter disease were associated with risk for delayed post-ICH dementia after 6 months [3]. Furthermore, it remains unclear whether post-ICH cognitive decline is truly secondary to the ICH itself or if haemorrhage is perhaps a symptom of an ongoing process that also led to cognitive decline. Neuroinflammation has been recognized and linked to the risk of dementia and it is increasingly recognized that a sustained immune response is a central feature of neurodegenerative disorders [4].

ICH triggers an inflammatory response that contributes to secondary brain injury, the clinical presentation and final outcome [5]. Experimental studies have suggested that ICH induces a cytokine-driven local acute-phase of inflammation, followed by a longer period of inflammation and oedema, resulting in a systemic inflammatory response syndrome (SIRS), which may lead to secondary brain damage [6,7,8].

In ICH, approximately 20% of patients develop SIRS during hospital stay [9]. SIRS presence correlates with stroke severity with detrimental short- and long-term functional outcome [10]. Locally, the inflammatory mediators fuel the inflammatory process surrounding the haematoma and increase perihaematomal oedema (PHO), exacerbating haematoma mass effect and increasing secondary brain tissue damage through ischaemic and inflammatory insults.

In the same context, the immune response evokes brain changes for organizing physiological and behavioural adaptations after an immune challenge. The brain structures involved in the induction of this inflammatory response behaviour are not well established, but there is evidence supporting the participation of the hypothalamus in the regulation of the inflammatory response [11]. A major function of the hypothalamus is to integrate sensory information and elicit adaptive changes in physiology and behaviour to achieve homeostasis [12].

Immune activation is a major challenge to homeostasis, so it should not be surprising that there is communication between the immune system and the hypothalamus. Clear documentation that such interactions exist has largely emerged from recent literature that has benefited from the identification and characterization of pro- and anti-inflammatory cytokines and has analysed their effect upon neural systems. It was clearly established that immune activation alters the turnover of classical neurotransmitters (e.g., noradrenaline, acetylcholine) and activates the HPA axis [13,14].

C-reactive protein (CRP) represents an important mediator of the acute-phase response to inflammation and its derivative monomeric CRP (mCRP), the cell- and tissue-associated biological active form produced during tissue damage [15]. CRP has been shown previously to relate to poor outcome after ICH, and preliminary results have shown that it can participate in local inflammatory response [16]. From this standpoint, pentameric CRP (pCRP) could represent an immune messenger acting as a mediator or regulator within the brain associated with general homeostasis.

In this pilot work, we describe the pattern of mCRP distribution after ICH in humans, and the transfer of mCRP in the central nervous system (CNS) and its neuronal expression within the hypothalamus in patients after ICH and in a hippocampal-mCRP-treated animal model. This work raises the possibility of intercommunication between distinct unaffected brain regions that could have damaging chronic effects on brain function, which underlies a sophisticated innate CNS immune system that is triggered by ICH.

## 2. Experimental Section

### 2.1. Human Brain Samples

Formalin-fixed, paraffin-embedded archived brain tissue blocks were selected from stroke patients (*n* = 11), and age-matched controls (*n* = 4), containing both lesional and peri-lesional areas. Autopsy material was obtained from the Clinic of Neurology (University of Medicine and Pharmacy Craiova). Death was due to the occurrence of secondary lesions, brain oedema and brain herniation, or cardiovascular arrhythmias. After the eventual death occurred, necropsy was performed. A written informed consent was obtained for each patient from the relatives or their caretakers. However, the ethical committee of the University of Medicine and Pharmacy Craiova did not require the study to be reviewed or approved because the material had previously been used for another study [17].

Further tissue samples (UK-Bristol Brain Bank, with ethical approval through application to https://mrc.ukri.org/research/facilities-and-resources-for-researchers/brain-banks/, and following their guidelines at https://mrc.ukri.org/publications/browse/human-tissue-and-biological-samples-for-use-in-research/; local approval was gained for the use of the tissue within the Manchester Metropolitan University LREC committee) had been collected within 4 h of death from the refrigerated bodies of 10 patients who showed clinical and histopathological evidence of both haemorrhagic stroke and AD/VaD. Samples were dissected into haematoma, infarcted (identified with 2,3,5-triphenyltetrazolium chloride), peri-infarcted/penumbra and normal looking unaffected tissue as previously described [17]. Peri-infarcted/penumbra tissue showed structural integrity but was characterized by oedema, altered morphology of the neurons (some showing changes of apoptosis), and angiogenesis. Tissue from the contralateral hemisphere served as a control. Samples were dissected into 2-mm diameter pieces and either frozen in liquid nitrogen at −70° or fixed in 10% buffered saline prior to paraffin embedding.

### 2.2. Aquaporin 4 (AQP4), Glial Fibrillary Acidic Protein (GFAP) and mCRP Localization by Immunofluorescence

Double immunofluorescence was performed after antigen retrieval. Briefly, after antigen retrieval by putting array slides for 40 min in 0.01 M of sodium citrate buffer at a pH of 6.0 and a temperature of 95 °C, and sections were allowed to cool to room temperature and were kept for 30 min in a 2% hydrogen peroxide solution. After blocking for 1 h in 10% goat serum, the slides were incubated sequentially first with the GFAP antibodies (1:50 in 1% Goat serum/0.1% Tween 20/1x PBS) for 18 h at 4 °C, followed after washings by the second primary antibody, rabbit anti-AQP4 (1:50, Santa Cruz, sc-20812) for 18 h at 4 °C. The next day, antigens were detected using a mix of goat anti rabbit CFL 488 and goat anti-mouse CFL 568 (1:100, Santa Cruz, Heidelberg, Germany) in 1% Goat serum/0.1% Tween 20/1x PBS for 4 h at room temperature. For AQP4/CRP double staining we used a mix of mouse anti-mCRP (1:200; clone M8C10,) and rabbit anti-AQP4 followed by a mix of secondary antibodies, goat anti-mouse histofine-HRP and goat anti-rabbit CFL 488. mCRP antigens were visualized by developing with tyramide Cy3 (Perkin Elmer, Rodgau, Germany). Sections were mounted in ProLong® Gold Antifade reagent with DAPI (Life Technologies, Karlsruhe, Germany) and slides were visualized on a Zeis LSM700 confocal microscope. Control cases were stained with a standard diaminobenzidine staining. Negative controls skipping the primary antibody showed no abnormal cross reactivity.

### 2.3. Counting of Colocalized mCRP/AQP4 and GFAP/AQP4 Positive Cells

Double-labelled cells were analysed in every 10th section in perihaematomal region as previously described [18]. To this end, a sequence of confocal counting images of 161 × 242 × 25 µm, spaced 0.1-µm apart across a 25-µm-thick section and covering 30% of the area of interest, were taken for fluorescently labelled cells. The relative mean number of double-labelled cells was then calculated by multiplying the number of cells per section times 3.3 (the counting boxes that were quantified covered one third of the area of each section) times the section interval of 10.

### 2.4. Animal Procedures

Mice from the inbred strain C57BL/6J (4-month-old; 25–30 g) were purchased to Janvier Labs, Le Genest-Saint-Isle, France. Animals were individually housed in Macrolon cages (Techniplast, Buguggiatta, Italy) with free access to food and water and maintained in a temperature-controlled room (22  ±  2  °C) with a 12-h light/12-h dark cycle. Animal handling, including surgical procedures and necropsies, was performed at the facilities of the Animal Unit of the University of Barcelona, Spain. The study was approved by the local animal experimentation ethics committee (Ref: DAAM-6991, CEEA, UB). All experiments were performed in accordance with ARRIVE Guidelines for the Care and Use of Laboratory Animals, and the Spanish guidelines/legislation concerning the protection of animals used for experimental and other scientific purposes and the European Commission Council Directive 86/609/EEC on this subject. All experimental protocols were approved by the above authority.

### 2.5. Hippocampal Injection of mCRP

Recombinant form of mCRP at the concentration of 1.75 mg/mL was produced in the laboratory of Dr. L.A. Potempa as previously described [19]. MCRP was delivered into the CA1 region of the mouse hippocampus by stereotactic surgery procedures. Four-month-old mice (*n* = 8 per group; 25–30 g) were anesthetized with 10 mg/kg of xylacine (Rompun 2%, Bayer, Leverkusen, Germany) i.p. and 80 mg/kg of ketamine (Ketolar 50 mg/ml, Pfizer, Alcobendas, Madrid, Spain) i.p. and placed in a stereotactic apparatus (David Kopf Instruments, Tujunga, CA, USA). Bilateral infusions of either an experimental agent solution or artificial CSF (NaCl 148  mmol/L, KCl 3  mmol/L, CaCl_2_ 1 mmol/L, MgCl_2_ 0.8  mmol/L, Na_2_HPO_4_ 0.8  mmol/L, NaH_2_PO_4_ 0.2  mmol/L) were performed into the CA1 area of the hippocampus. Each mouse received 3.5 µg of mCRP. Injections were performed at a rate of 1  μL/min at coordinates relative to Bregma of −2.0  mm A/P, ±  1.2  mm M/L, −1.5  mm V/D. One microliter of the testing solutions was delivered to the application point with a 25-gauge stainless steel cannula (Small Parts Inc., Miami, FL, USA) connected to a Hamilton syringe through a Teflon tube. The syringe was attached to a microinfusion pump (Bioanalytical systems Inc., West Lafayette, IN, USA). The cannula was left in position for 5 min after delivery to prevent the solution from surging back. All mice were visually inspected and weighed on a regular basis throughout the 4-week study to control for their general health status.

### 2.6. Histological Analysis of Mice Brain Tissue

Mice were anesthetized as described above and transcardially perfused with 100  mM of phosphate buffer (PB, pH 7.4) containing 0.1 mg/ml of heparin (Mayne Pharma, Salisbury, South, Australia) followed by 4% paraformaldehyde in PB. Brains were removed and post-fixed overnight in cold paraformaldehyde, rinsed with cold PB and then dehydrated in a graded ethanol series, cleared in xylene and embedded in paraffin. Serial sections were cut throughout the brain at 5  μm, and immunohistochemistry (IHC) was carried out at 1-mm intervals throughout the brain details described below in the section on immunohistochemistry (animals *n* = 8 per group) in order to determine expression and localization of mCRP.

### 2.7. Immunohistochemistry of Mice Brain Tissue

Immunohistochemistry was used to assess the distribution of mCRP (mouse antihuman mCRP-specific antibodies 8C10) After incubation with primary antibodies for 1  h at room temperature (1:100), sections were washed and then incubated with the appropriate biotinylated secondary antibodies (1:50), followed by diaminobenzidine staining. Nuclei were stained with Vector Nuclear Fast Red. Images were captured with Nikon 80i Digital Microscope using Nis Elements 3.21 software with multichannel capture option. Negative control slides were included where the primary antibody was replaced with PBS. Vector ABC kits were used for all IHC and the Vector mouse on mouse (M.O.M) was used when applying mouse primary CRP antibodies to the murine brain sections with mouse secondary.

### 2.8. Statistical Analysis

The main effects of cellular localization and colocalizations were evaluated by ANOVA followed by Bonferroni post-hoc analyses using GraphPad Software (Version 7.0, San Diego, CA, USA). The between-groups analysis was performed using post-hoc tests for multiple comparisons. The level of significance was set at *p* ≤ 0.05.

## 3. Results

### 3.1. Neuropathological Macroscopic Examination

At necropsy, general neuropathological evaluation confirmed focal brain matter softening, discoloration, petechial haemorrhages or ventricular blood inundation in more recent lesions, and yellowish gliotic areas and cavitations in organized lesions. Microscopically, a classical array of neuronal eosinophilia, shrunken pericarions, liquefactive necrosis, reactive gliosis, perilesional neuronal loss and cortical vacuolation characterized ischemic lesions. Moderate vasculitis, petechial or large haemorrhages and perilesional cortical vacuolation characterized haemorrhagic transformations or per primam haemorrhagic events. Peri-infarcted tissue showed structural integrity but was characterised by oedema, altered morphology of the neurons (some showing changes of apoptosis) and angiogenesis.

### 3.2. Immunohistochemical Analysis

#### 3.2.1. AQP4/mCRP Localization in the Perihaematomal Region

**Figure 1:**
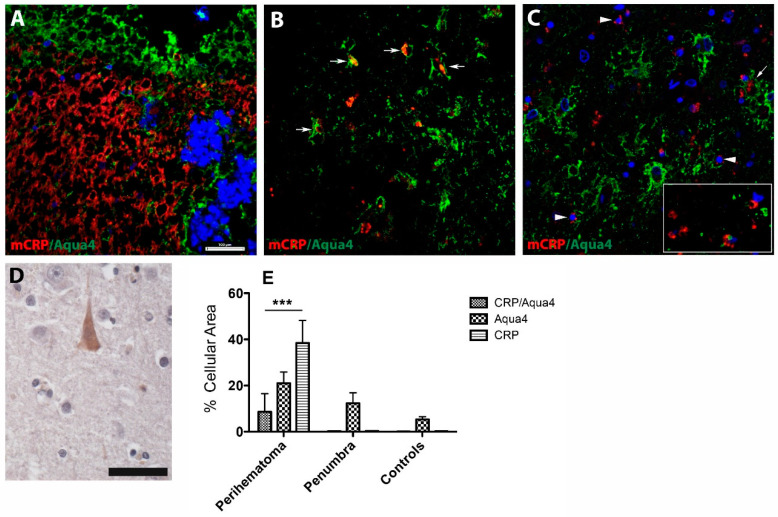
Monomeric C-reactive protein (mCRP) and aquaporin 4 (AQP4) expression pattern in the perihaematomal area. (**A**) In the haemorrhagic stroke area of early deaths (<48 h), mCRP was seen in numerous microvessels, while AQP4 immunoreactivity was localized adjacent to the mCRP staining and lined up with the haemorrhagic core. However, with increasing post-stroke survival time, mCRP immunostaining was detected in the lumen of microvessels expressing AQP4 (**B**, arrows) and some parenchymal brain cells and some stroke-affected neurons in the surrounding areas (**C**, arrowheads) and the lumen of large blood vessels (**C**, arrow) and brain capillaries (**C**, inset). In the normal areas of the brain, there was a weak cytoplasmic expression of mCRP in cortical neurons (**C**). A quantification of the cellular area covered by mCRP- and AQP4-positive cells revealed that mCRP-positive cell staining was predominant in the perihaematomal area, while AQP4-positive structures populated areas in the perihaematomal area followed by penumbra and, to a lesser extent, in controls. Occasionally, in the normal areas of the brain, there was a weak cytoplasmic expression of mCRP in cortical neurons (**D**). Colocalized mCRP/AQP4 cells were found only in the perihaematomal area (**E**). Scale bars are 100 µm and 20 µm for DAB staining. *** *p* = 0.001. In the figure legend, CRP indicates mCRP; Aqua4, AQP4.

Using double immunofluorescent staining, we found that mCRP was abundantly seen in the perihaematomal region of patients who died early (2 days) after stroke (Figure 1A, red).

At this early stage, AQP4 immunoreactivity was localized adjacent to the mCRP staining and lined up with the haematoma core (Figure 1A, green). Further from the perihaematomal region, however, mCRP was detected in the lumen of microvessels expressing AQP4 (Figure 1B, arrows). However, with increasing post-stroke survival time, mCRP immunostaining was associated with some parenchymal brain cells, some stroke-affected neurons in the surrounding areas (Figure 1C, arrowheads) and the lumen of large blood vessels (Figure 1C, arrow), as well as brain capillaries (Figure 1C, inset). Occasionally, in the normal areas of the brain, there was a weak cytoplasmic expression of mCRP in cortical neurons (Figure 1D). A quantification of the cellular area covered by mCRP- and AQP4-positive cells revealed that mCRP-positive cells were predominant in the perihaematomal area, while AQP4-positive structures populated areas in the perihaematomal area followed by penumbra and, to a lesser extent, in controls. Colocalized mCRP/AQP4 cells were found only in the perihaematomal region (Figure 1E).

#### 3.2.2. GFAP/AQP4 Localization in the Perihaematomal Region

**Figure 2:**
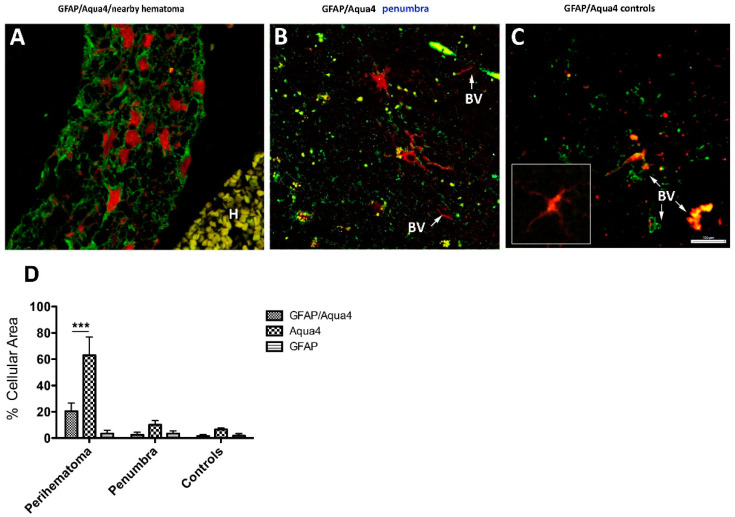
Glial fibrillary acidic protein (GFAP) and aquaporin 4 (AQP4) expression pattern in the perihaematomal area Many reactive astrocytes expressing AQP4 were detected in the proximity of large haematomas areas (**A**). Perihaematomal areas were populated mostly with reactive astrocytes on a background of diffuse aquaporine4 immunostaining (**B**, green). In controls, astrocytes and AQP4 were codistributed on blood vessels and astrocytes having a normal morphology (**C** and inset). A quantification of the cellular area covered by GFAP- and AQP4-positive cells revealed that AQP4-positive cells were predominant in the perihaematomal area, followed by colocalized GFAP/AQP4 structures (**D**). *** *p* = 0.001. In the figure legend, Aqua4 indicates AQP4.

A high density of reactive astrocytes expressing AQP4 was detected in the proximity of large haematoma areas (Figure 2A).

Metabolic penumbra areas were populated mostly with reactive astrocytes on a background of diffuse AQP4 immunostaining (Figure 2B, green). In controls, astrocytes and AQP4 were codistributed on blood vessels and astrocytes having a normal morphology (inset) (Figure 2C). A quantification of the cellular area covered by GFAP- and AQP4-positive cells revealed that AQP4-positive cells were predominant in the perihaematomal area, followed by colocalized GFAP/AQP4 structures (Figure 2D).

#### 3.2.3. Haematoma, Peri-Haematoma and Hypothalamus mCRP Staining

**Figure 3:**
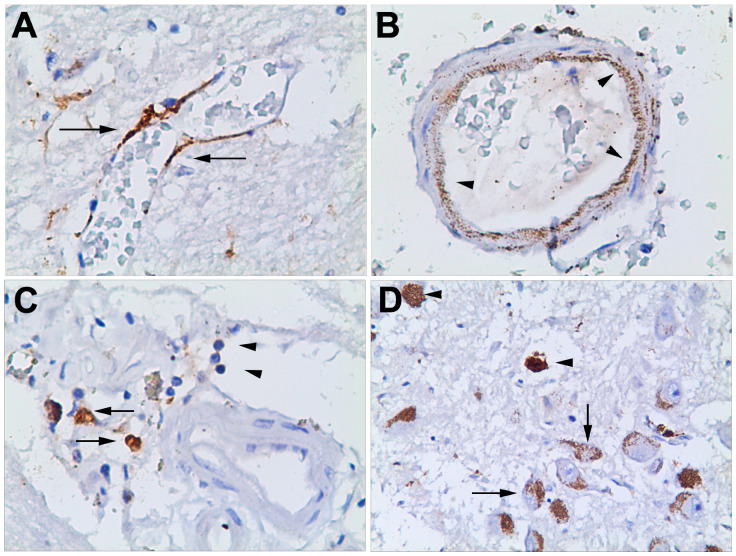
In some samples, within a certain stage (<24 h) of haemorrhage development, strong staining was observed within capillary endothelial cells, especially within the haemorrhagic area (**A**,**B**; arrows). In severely damaged areas, mCRP was more strongly observed (**C**, arrows), associated within the infiltrating macrophages and inflammatory cells (**C**, arrowheads). We also detected clusters of neurons loaded with mCRP (**D**, arrows) along with scattered lipofuscin-like deposits (**D**, arrowheads).

Concerning ICH patients, the severity of the lesion did not affect neuronal staining, which is clearly visible in all of our samples. In the very early stages of haemorrhage (<6 h), there was no staining of mCRP in brain samples. Thereafter, within haemorrhagic stroke areas, there was strong staining in neurons throughout most of the cortical layers. This staining was independent of the level of damage apparent to the neurons and could be seen across type I through type V/VI neurons. Neurons were the main cellular type in the stroke brain stained for mCRP. This was independent of whether the areas were close to or at the edge of the haematoma. In the early stages of haemorrhage, positively stained macrophages can be seen, but there was no staining within the blood vessels (either macrovessels or microvessels or capillaries). In some samples, within a certain stage of haemorrhage, (>24 h) there was strong staining within capillary endothelial cells, especially within the haemorrhagic (Figure 3A,B; arrows).

Where there was more severe damage to the tissue, mCRP was more strongly observed (Figure 3C, arrows) associated within the infiltrating macrophages and inflammatory cells (Figure 3C, arrowheads). Finally, we detected clusters of neurons loaded with mCRP (Figure 3D, arrows), along with scattered lipofuscin-like deposits (Figure 3D, arrowheads).

#### 3.2.4. mCRP Distribution after Local Delivery to Mice

**Figure 4:**
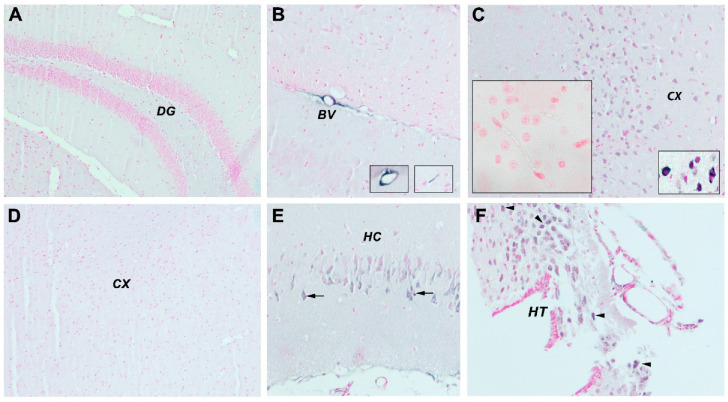
In our stereotactically mCRP-injected murine model, we observed the pattern of mCRP distribution after 4 weeks. We found no evidence of mCRP staining in control mice (**A**,**D**), and a strong hippocampal staining of neurons in mCRP injected mice (**E**) adjacent to the injection site. (**C**) Many medium and small cortical vessels became positively stained for mCRP (**B**), and hypothalamic neurons became positively stained in the cytoplasm of mCRP-treated mice (**F**). The inset in (**C**) shows the isotype IgG control. Abbreviations: DG indicates dentate gyrus of the hippocampal formation, BV, blood vessels; CX, cerebral cortex; HC, hippocampus; HT, hypothalamus.

We previously showed that stereotactic injection of mCRP into the hippocampus of mice caused dementia-like deficiencies in behavioural and cognitive performance concomitant with neurodegenerative cellular protein expression and morphology within 4 weeks of treatment [15]. Interestingly, we noticed that only in mCRP-injected mice, apart from the local hippocampal neuronal staining adjacent to the injection site (Figure 4E), many small cortical microvessels became positively stained for mCRP (Figure 4B and insets), indicating a possible spread mechanism throughout the cortex and beyond. In addition, neurons in the cerebral cortex (Figure 4C) and in the distant hypothalamus (Figure 4F) became positively stained in the cytoplasm, while control mice did not express it in the hippocampus (Figure 4A) or cortex (Figure 4D).

## 4. Discussion

We previously identified increased tissue localization of mCRP in samples of spontaneous intracerebral haemorrhage autopsy brains [17]. mCRP induces strong inflammation and may contribute to an impaired regenerative and reparative potential of the tissue after stroke. In this pilot study, we provided evidence that may suggest that some stress reaction induced by ischaemic/haemorrhagic events leads primarily to the neuronal expression of mCRP. This also further suggests that mCRP may function as a mediator of stress response after ischaemia/haemorrhage in the brain. The second line of response/possibility would be penetration via the capillary wall of monocytes/macrophages or local inflammatory activation of the microglia once the lesion is more advanced. Indeed, positive staining in macrophages/microglia was in more advanced lesions. Finally, mCRP could contribute to late degeneration, affecting several distant brain areas.

All of the above is in line with a previous report on the expression of mCRP in human stroke brains [20]. The strong neuronal staining was linked to the presence of microbleeds in the brain. Patients with more than four microbleeds on CT/MRI who confirmed later pathology had more abundant expression of neuronal mCRP. This is interesting, as increasing numbers of microbleeds have been associated with worse cognitive impairment [21]. This also further links mCRP with dementia. ICH survivors appear to develop cognitive impairment at high rates. Thus, early incident dementia after ICH is strongly associated with haematoma size and location, while delayed dementia, which is frequent among ICH survivors, is not prominently associated with acute ICH characteristics [3]. Because neuronal expression requires more time to appear, neuronal expression of mCRP primarily suggests that mCRP may function not only as a mediator of stress response after ICH, but also as an important mediator of neurodegeneration, being related to chronicity and longer time of evolution of microbleeds. These findings also suggest the existence of heterogeneous biological mechanisms, accounting for the delayed cognitive decline among ICH survivors.

Regarding endothelial cell mCRP expression, it seems that it is driven by pure ischaemia and appears in the early stages of ischaemic stroke. Any reactive astrocytes expressing AQP4 and mCRP were detected in the proximity of large haematomas areas, strongly suggesting that these are key players facilitating the entry of water in the perihaematomal area and contributing to brain oedema, a major cause of mortality in stroke patients.

Furthermore, because AQP4 is a part of a brain-wide network of channels, collectively known as the glymphatic system [22], which permits cerebral-spinal fluid from outside of the brain to wash away toxic proteins, it may play a role in efficiently clearing mCRP deposition in perihematomal zones and reducing tissue damage. To our knowledge, this is the first report of a codeposition of mCRP and AQP4 in brain tissue after ICH. Furthermore, the loss of perivascular AQP4 localization may be a factor that renders the aging brain vulnerable to the toxic aggregation of proteins, such as amyloid-β, in neurodegenerative conditions such as AD.

Circulating levels of pCRP are also associated to the development of dementia, the conversion of mild cognitive impairment in dementia and the development of other neurodegenerative diseases, as well as activate neurodegenerative pathways that may be associated with dementia [2,3,22,23]. Furthermore, the implication of mCRP in neuroinflammation has been established over the last few years [16,24]. mCRP is found within the damaged brain tissue after stroke and has the capacity to perpetuate inflammation, as well as activate neurodegenerative pathways that may be associated with dementia [24,25].

The transformation of the circulating pCRP to its proinflammatory structural isoform and further dissociation to mCRP and thus activation of the CRP system occurs on necrotic, apoptotic and ischemic cells; regular β-sheet structures, such as β-amyloid; the membranes of activated cells (e.g., platelets, monocytes and endothelial cells) and/or the surface of microparticles, the latter by binding to phosphocholine. mCRP can cause the activation of platelets, leukocytes, endothelial cells and complements [26]. mCRP is now proposed as an important pathogenic factor, potentially destabilizing atherosclerosis and predisposing patients to myocardial infarction [10,27]. Therefore, taking the evidence together, the inhibition of pCRP dissociation represents a promising, novel anti-inflammatory therapeutic strategy.

Our hypothesis about the hypothalamus as a brain sensor connected with peripheral inflammation is supported by work of Fang and Yujie [10]. They showed that when haemorrhagic lesions existed in the parietal cortex (resulting in paralysis of the contralateral hand), the sympathetic centre of the posterior lateral nucleus of the hypothalamus demonstrated compensatory excitement, in this case, often leading to tachyarrhythmia and sudden death. In addition, we suggest that mCRP, even following local injection as seen in our mouse model or following leakage from existing cortical vessels in haemorrhagic stroke, could be transported through the existing microvascular structures to distant locations including the hypothalamus as seen using our murine-hippocampal-mCRP-injected model. Based on these observations, peripheral production of CRP in the liver can be seen as an important sensor for systemic inflammation, communicating the inflammatory status of the body via the autonomic nervous system to the brain and, finally, being an essential target of the inflammatory reflex [13].

## 5. Conclusions

mCRP is abundantly expressed in the brain after haemorrhagic stroke, impacting the pathophysiological development of the metabolic penumbra directly, as well as indirectly, as the microcirculatory system appears to be able to carry it throughout the cortex and as far as the hypothalamus, allowing for long-distance effects and damage through its capacity to induce inflammation and degenerate neuronal perivascular compartments.
